# Consumption of aquatic products and meats in Chinese residents: A nationwide survey

**DOI:** 10.3389/fnut.2022.927417

**Published:** 2022-07-22

**Authors:** Qihe Wang, Sana Liu, Huijun Wang, Chang Su, Aidong Liu, Liying Jiang

**Affiliations:** ^1^Department of Nutrition Division I, China National Center for Food Safety Risk Assessment, Beijing, China; ^2^Department of Public Nutrition and Nutrition Policy, Chinese Center for Disease Control and Prevention, Beijing, China; ^3^Shanghai Key Laboratory of Molecular Imaging, Jiading Central Hospital, Shanghai University of Medicine and Health Sciences, Shanghai, China

**Keywords:** aquatic products, meats, consumption, socio-economic status, China

## Abstract

**Objective:**

To provide the most recent national estimates for the consumption of aquatic products and meats among Chinese residents.

**Methods:**

This study was conducted in 14 provinces of China, using a multi-stage stratified random cluster sampling method and a population-proportional sampling procedure. Aquatic products and meats consumption was measured by a 3-day, 24-h dietary recall. Chinese residents aged 3 years and above (*n* = 24,106) completed a face-to-face dietary interview.

**Results:**

The average daily consumption of meat and aquatic products for the all-aged population was 70.9 g and 48.0 g, respectively, which aligned with Dietary Guidelines (40–75 g/d) for Chinese Residents (2016). On the one hand, intake of aquatic products among Chinese people was relatively insufficient, especially for adolescents and elder people (<40 g/d). On the other hand, males, mainly aged 19–60, generally consumed too much meat (>80 g/d), and 19–44 grouping consumed more than 70 g/d of red meat. Besides, urban residents and individuals with higher socioeconomic status (SES) have exhibited comprehensively healthy dietary preferences than rural ones and those with a lower SES do. Women and the higher SES group tend to be closer to the dietary guidelines for the Chinese.

**Conclusions:**

The consumption of meat and aquatic products varied with age, gender, region and SES. Detecting patterns in consumption is particularly relevant for policy makers, researchers and health professionals in the formulation of dietary recommendations and estimating potential health outcomes.

## Introduction

Increased prevalence of diet-related chronic diseases has become a public health problem in China, a country undergoing a nutrition transition. A healthy dietary pattern could significantly reduce the risk of overweight, obesity, metabolic syndrome and diabetes, and benefit health throughout all stages of life (1–4). In Asian diets, dietary carbohydrates are the major source of energy (5). Over the past decades, rapid economic development has introduced remarkable changes in diet patterns in China. People have come to realize the vital role of protein in the human body. A considerable increase has been observed in demand for meats and aquatic products. A growing body of research worldwide has examined the consumption pattern of these two food categories and their association with diet-related health and diseases (6–8). Previous studies have demonstrated that a high intake of red meat, especially processed meat, is related to an increased risk of hyperlipidemia, hypertension, osteoporosis, stroke, cancer, and all-cause deaths (9–15). White meats (such as poultry and aquatic products), which contain high-quality protein and a wide range of essential micronutrients with relatively lower saturated fats and cholesterol, are considered a healthier choice (16).

Indeed, present dietary recommendations for the prevention of diet-related chronic diseases have promoted lower intake of red and processed meat and higher intake of plant-based foods. And countries worldwide have introduced dietary guidelines for their residents. The latest Guidelines for Australian Adults advise people to limit their intake of foods containing saturated fat (17). However, Lee et al. suggested that there was a lack of association between dietary saturated fatty acids and non-communicable diseases (18, 19), which contradicted with the advice to limit saturated fatty acids consumption from the American Heart Association. Dietary recommendations need further study and demonstration to promote human health. At the same time, the 2015–2020 Dietary Guidelines for Americans recommended the consumption of a variety of protein foods, including seafood, lean meats, and poultry (20). China also introduced a new version of the Dietary Guidelines for Chinese Residents (DGC) in the form of the Chinese Food Pagoda in 2016, intending to help residents adopt healthful lifestyles and improve fitness (21).

So far, nutrition studies are mainly carried out in scientific research institutions in China, limiting the diversity and comprehensiveness of samples. The Chinese Government attaches great importance to national health and conducts a nationwide survey of residents' food consumption. Although China's national bureau of statistics (NBS) collects annual statistics on the food consumption of residents, the information on the distribution of food consumption among different groups of people (demography or sociology) is limited. Obviously, healthy choices do not rely on single food groups only but on the overall pattern and eating style. People with different gender, age groups, regions and socioeconomic statuses (SES) have different food consumption situations. Individuals with higher nutritional awareness, higher education level and higher economic status tend to choose different animal meat consumption. Moreover, the local ecological environment determines the availability of food. China has a vast territory and rich products. There are great differences in the dietary preferences and styles of residents in various regions. This presents a gap in knowledge about which groups would eat more red meat or which populations may have healthier meat consumption patterns. Therefore, it is necessary to conduct a nationwide survey of aquatic products and meat consumption among Chinese residents.

Using a representative nationwide sample of Chinese residents, the present study aimed to assess the consumption of aquatic products and meats, and further explore the difference in dietary behavior among different gender, age groups, regions and SES groups. This study may shed light on the understanding of food consumption and dietary pattern in China that would guide further scientific research and policy-making.

## Materials and methods

### Study design and participants

In present study, data were from the Chinese Food Consumption Survey (CFCS), which was a cross-sectional national survey conducted in China during July to October 2019. The participants were aged ≥ 3, Chinese-speaking, and living in private households. This survey was a collaborative project among the China National Center for Food Safety Risk Assessment (NFSA), the Chinese Center for Disease Control and Prevention (CDC), and Community Services Centers. The survey was designed to collect information on individuals' consumption of alcohol, beverages, meats, aquatic products, and processed foods, as well as health behavior, and to further examine how potential risk factors affect people's health.

The sample was collected using a multistage stratified random sampling procedure and a population-proportional sampling (PPS) technique in 14 provinces, autonomous regions, and municipalities in China, including Hebei, Inner Mongolia autonomous region, Liaoning, Heilongjiang, Jiangsu, Zhejiang, Jiangxi, Shandong, Hubei, Guangdong, Chongqing, Guizhou, Yunnan, and Gansu. Thirty-one sites were selected randomly from cities or counties in the above provinces. Three towns/streets in each survey site, two villages/communities in each town/street, and fifty households in each village/community were selected. Each survey site included at least 300 households and 900 respondents. Inhabitants aged 3 years and older who had lived in the neighborhood for at least 6 months were identified. 24,106 participants were recruited to the study. The response rate was above 85%.

### Data collection

Household conditions (family population, annual per capita income) and individual demographics (age, gender, nationality, marital status, education, and occupation), were obtained using a self-reported questionnaire. Trained staff then conducted a face-to-face interview using a standard questionnaire at the home of the participants. Dietary assessment survey was based on a combination of data collected using a validated food frequency questionnaire (FFQ) and a self-reported, 3-day, 24-h recall at the individual level. i) The 3-day, 24-h retrospective survey method, aimed to estimate individual food consumption based on the weight of food purchased and the percentage of that day's consumption within 24 h of the survey date. Considering daily variations in food intake, the participants were interviewed on two consecutive weekdays and 1 weekend day. The investigation of aquatic food items mainly concerned fish, crustaceans, mollusks, and alga; while that of meat items included livestock and poultry. ii) The FFQ was used to measure the food consumption of samples in the last 12 months and the questionnaire was administered by an interviewer. The consumption frequency survey covered fresh and processed aquatic products, animal offal, traditional Chinese processed meats, and hand-made and industrially processed meat products over the last 12 months. As the survey mainly depended on the memory of respondents to recall and describe their meals, memory alone would bring some problems, such as blurred and indistinct memory. Therefore, we used a multi-pass technique to overcome the issue. First of all, the investigators of this study must undergo strict professional training and be able to guide subjects to finish their interviews even in some specific situations, aiming to avoid omissions. The investigator asked the subjects about their meals within 24 h in the household survey every day to avoid recalling their diets after a long time. Meanwhile, for the aged under 12 and the elderly, information would be collected by asking the guardian. Moreover, after checking the collected data of each sample, the investigators upload the survey data to the network database through the smart tablet every day. After online verification and timely correction of the uploaded data of each survey site by the data inspectors, the data was finally reported to the national working group.

All respondents aged 3 and older with reliable dietary data were included and appropriate weighting factors were applied to adjust for differential probabilities of selection and non-response. Household food consumption data were used to estimate the consumption of meat and aquatic products. All participants were fully informed in detail about the objective and the procedure before enrolling in the study and signed a written consent form. For children younger than 12 years, parents or primary caregivers were asked to recall the children's food consumption and signed informed consent. All minors (below the age of 18) were investigated with the informed consent of their parents or guardians (parent or legal guardian consent was obtained on behalf of all minors). The surveys were approved by the Ethical Review Committee of China National Center for Food Safety Risk Assessment.

### Categorization of meats and aquatic products

“Fish” refers to fresh-water fish and sea-fish. “Crustaceans” refers to shrimp and crabs. “Mollusks” refers to shellfish, snails etc. “Livestock meat” mainly refers to pork, beef, and mutton, which includes muscle meat only, not offal. “Poultry” refers to chickens, ducks, and other poultry. “Processed meat” includes sausages, bacon, ham, nuggets, salami, and other fermented meats. “Aquatic processed products” refers to smoked, pickled, and industrially processed products. All organ and offal meat were reported together because of the low frequency of consumption at the population level. “Traditional Chinese processed meats” refers to smoked, soy sauce, and pot-stewed meat. “Hand-made and industrially processed meat products” refers to cured meat, sausages, pork floss, canned meat, etc.

### Statistical analysis

We categorized the subjects into five age groups: 3–18, 19–30, 31–45, 46–60, and ≥61 years, and also performed further analyses stratified by gender. To describe the social status of the participants, a social class-index (SEI) was introduced based on the monthly income of the household, education level and the employment status of the principal earner in the household to evaluate the SES of Chinese residents (22).

Description of the residents' aquatic products and meats consumption by age and gender was presented as an arithmetic mean and 95% confidence interval (CI). Pairwise comparisons of the means across groups were tested using one-way classification analysis of variance (ANOVA), and a *post hoc* multiple comparison was performed using a least significant difference-*t* test. A level of α < 0.05 was considered statistically significant and all statistical significance tests were two-sided. All analyses were completed using Stata version 13.0 (StataCorp, College Station, TX, USA).

## Results

The demographical characteristics of the sample is consistent with that of official national statistics in China (Statistical Bulletin of the National Economic and Social Development of the People's Republic of China in 2017). Briefly, the number of households involved in the survey are over 4,200. Among the 24,106 participants who completed the retrospective dietary interview, 11,986 were men (49.7%) and 12,120 were women (50.3%). The mean age was 40.2 years for men and 40.7 years for women. The characteristics of the participants are shown in Table 1.

**Table 1:** Characteristics of the participants in the retrospective dietary interview (*n* = 24,106).

	**Male** **(*****n*** = **11,986)**	**Female (*****n*** = **12,120)**
Age		
3–18	2,127 (17.8%)	1,920 (15.8%)
19–30	1,517 (12.7%)	1,635 (13.5%)
31–44	2,954 (24.6%)	2,957 (24.4%)
45–59	3,442 (28.7%)	3,657 (30.2%)
≥60	1,946 (16.2%)	1,951 (16.1%)
Area		
Urban	4,257 (35.5%)	4,649 (38.4%)
Rural	7,729 (64.5%)	7,471 (61.6%)
Career		
Student	1,567 (13.1%)	1,438 (11.9%)
Housework	670 (5.6%)	2,649 (21.9%)
Unemployed	383 (3.2%)	401 (3.3%)
Retired	941 (7.9%)	970 (8.0%)
Government	416 (3.5%)	268 (2.2%)
Technician	1,157 (9.6%)	753 (6.2%)
Administrator	478 (4.0%)	570 (4.7%)
Service staff	1,112 (9.3%)	1,209 (10.0%)
Producer	2,787 (23.2%)	2,270 (18.7%)
Operator	606 (5.0%)	138 (1.1%)
Soldier	20 (0.2%)	3 (0.0%)
Preschooler	621 (5.2%)	547 (4.5%)
Other	1,228 (10.2%)	904 (7.5%)
Education		
Pre-school	632 (5.3%)	557 (4.6%)
Illiteracy	243 (2.0%)	824 (6.8%)
Primary school	2,847 (23.8%)	3,166 (26.1%)
Junior school	4,399 (36.7%)	4,095 (33.8%)
High school	2,292 (19.1%)	2,016 (16.6%)
Junior college	845 (7.0%)	802 (6.6%)
University & above	728 (6.1%)	660 (5.5%)
Household income (yuan/year)		
None applicable	585 (4.9%)	591 (4.9%)
≤4,999	1,636 (13.6%)	1,742 (14.4%)
5,000–9,999	2,087 (17.4%)	2,081 (17.2%)
10,000–14,999	2,331 (19.4%)	2,305 (19.0%)
15,000–19,999	1,421 (11.9%)	1,424 (11.7%)
20,000–24,999	1,190 (9.9%)	1,208 (10.0%)
25,000–29,999	750 (6.3%)	772 (6.4%)
30,000–34,999	715 (6.0%)	705 (5.8%)
35,000–39,999	356 (3.0%)	359 (2.9%)
≥40,000	915 (7.6%)	933 (7.7%)
SES (%)	Male (*n* = 8,176)	Female (*n* = 8,834)
Low	3,788 (46.3%)	3,378 (38.2%)
Medium	2,486 (30.4%)	3,983 (45.1%)
High	1,902 (23.3%)	1,473 (16.7%)

### Consumption of aquatic products and meats

On average, participants consumed fish almost twice a week, while they consumed crustaceans and alga no more than once a week. Participants had relatively low frequencies of the consumption for mollusks and processed aquatic products, with an average of nearly once a month. In terms of meat products, the participants consumed processed meat (excluding barbecued meat) at a frequency of 3 times per month. Comparatively, the participants were less likely to consume animal offal, with an average frequency of lower than once a month. In general, the consumption frequency of barbecued meat was low. The consumption of meat per day was 70.9 g/d (95% CI: 70.0, 71.7) and that of aquatic products was 48.0 g/d (95% CI: 47.2, 48.8).

Consumption of aquatic products is presented in Table 2. The participants mainly consumed fish (70–80%), followed by crustaceans (15–20%). The consumption of other aquatic products was somewhat low. Younger adults and middle-aged groups (19–60 years) presented a higher level of consumption of aquatic products compared to older adults. The 31–44 age group consumed the highest amount (55.7 g/d) of total aquatic products. Children and adolescents (3–18 years) consumed the least amount (32.1 g/d). The difference of consumption between men and women was 7.6 g/d.

**Table 2:** Daily aquatic products consumption by gender and age groups using 24 h recalls (g/d) (*n* = 24,106)^*^.

**Group**	**Gender**	**3–18 years**	**19–30 years**	**31–44 years**	**45–59 years**	**60–99 years**	**Average**
		**Mean (95% CI)**	**Mean (95% CI)**	**Mean (95% CI)**	**Mean (95% CI)**	**Mean (95% CI)**	**Mean (95% CI)**
Alga	Male	0.129 (0.059, 0.198)	0.134 (0.062, 0.205)	0.191 (0.108, 0.274)	0.255 (0.170, 0.340)	0.224 (0.127, 0.322)	0.196 (0.158, 0.235)
	Female	0.121 (0.061, 0.181)	0.211 (0.114, 0.307)	0.309 (0.162, 0.456)	0.204 (0.139, 0.268)	0.191 (0.108, 0.274)	0.215 (0.169, 0.261)
Crustaceans	Male	7.21 (6.27, 8.15)	10.3 (8.75, 11.8)	12.6 (11.4, 13.8)	9.80 (8.90, 10.7)	7.61 (6.51, 8.71)	9.73 (9.23, 10.2)
	Female	6.94 (5.96, 7.92)	9.18 (7.88, 10.5)	11.5 (10.3, 12.8)	7.97 (7.22, 8.72)	6.51 (5.48, 7.54)	8.61 (8.14, 9.08)
Fish	Male	24.9 (23.2, 26.6)	40.3 (37.6, 43.0)	44.2 (42.1, 46.3)	44.9 (43.0, 46.8)	38.2 (35.9, 40.5)	39.5 (38.5, 40.5)
	Female	21.7 (20.0, 23.3)	36.4 (33.5, 39.2)	36.9 (35.1, 38.7)	37.0 (35.4, 38.7)	30.8 (28.9, 32.8)	33.5 (32.6, 34.3)
Mollusks	Male	1.56 (1.12, 2.00)	2.23 (1.63, 2.83)	3.06 (2.58, 3.54)	2.37 (1.99, 2.75)	2.28 (1.77, 2.78)	2.36 (2.15, 2.57)
	Female	1.48 (1.16, 1.79)	1.62 (1.23, 2.02)	2.63 (2.24, 3.01)	2.03 (1.71, 2.35)	1.48 (1.14, 1.83)	1.95 (1.78, 2.11)
Total	Male	33.8 (31.6, 35.9)	52.9 (49.3, 56.4)	60.1 (57.3, 62.8)	57.3 (54.9, 59.8)	48.3 (45.3, 51.2)	51.8 (50.6, 53.0)
	Female	30.2 (28.1, 32.3)	47.4 (43.9, 50.8)	51.3 (48.9, 53.8)	47.3 (45.2, 49.3)	39.0 (36.5, 41.5)	44.2 (43.1, 45.3)
Total		32.1 (30.6, 33.6)	50.0 (47.5, 52.5)	55.7 (53.9, 57.5)	52.1 (50.5, 53.7)	43.6 (41.7, 45.6)	48.0 (47.2, 48.8)

* *Keeping three valid digits*.

The consumption of meat and meat products for the participants is shown in Table 3. Pork was the most consumed category, which constitutes 2/3 of the total consumption, followed by poultry (about one fifth); beef and mutton were the least consumed. Meat consumption varied by age and gender. Children, adolescents, and the elderly over 60 years old consumed about 60 grams meat per day, whereas younger adults and middle-aged people consumed more than 75 g/d meat per day on average. Males had higher consumption of meat than females with a difference of 13.3 g/d, and the certain difference could be mainly detected in red meat.

**Table 3:** Daily meat consumption by gender and age groups using 24 h recalls (g/d) (*n* = 24,106)^*^.

**Group**	**Gender**	**3–18 years**	**19–30 years**	**31–44 years**	**45–60 years**	**61–99 years**	**Average**
		**Mean (95% CI)**	**Mean (95% CI)**	**Mean (95% CI)**	**Mean (95% CI)**	**Mean (95% CI)**	**Mean (95% CI)**
Beef	Male	4.63 (3.86, 5.39)	7.59 (6.40, 8.77)	6.80 (6.08, 7.52)	5.55 (4.92, 6.19)	2.91 (2.37, 3.46)	5.53 (5.19, 5.86)
	Female	2.87 (2.34, 3.41)	5.43 (4.53, 6.33)	5.04 (4.46, 5.63)	4.10 (3.56, 4.64)	2.42 (1.95, 2.89)	4.05 (3.77, 4.32)
Mutton	Male	2.70 (1.98, 3.42)	5.09 (3.96, 6.23)	3.92 (3.23, 4.62)	4.10 (3.41, 4.80)	2.28 (1.71, 2.84)	3.64 (3.30, 3.98)
	Female	2.15 (1.59, 2.71)	2.84 (2.17, 3.50)	3.01 (2.44, 3.58)	2.75 (2.27, 3.23)	1.37 (1.00, 1.73)	2.51 (2.26, 2.75)
Pork	Male	42.0 (40.3, 43.7)	56.5 (54.0, 59.1)	60.3 (58.3, 62.3)	56.0 (54.4, 57.6)	48.1 (46.1, 50.0)	53.3 (52.5, 54.2)
	Female	40.2 (36.4, 43.9)	46.4 (44.2, 48.6)	48.3 (46.8, 49.8)	46.4 (45.0, 47.7)	41.0 (39.4, 42.7)	45.0 (44.1, 45.9)
Poultry	Male	13.9 (12.7, 15.2)	15.7 (14.1, 17.2)	17.2 (16.0, 18.4)	15.3 (14.2, 16.3)	11.2 (10.2, 12.3)	14.9 (14.4, 15.5)
	Female	11.7 (10.6, 12.9)	15.0 (13.6, 16.5)	14.4 (13.3, 15.4)	12.1 (11.3, 12.9)	9.14 (8.20, 10.1)	12.5 (12.0, 13.0)
Total	Male	63.2 (60.5, 66.0)	84.9 (80.9, 88.9)	88.3 (85.3, 91.3)	80.9 (78.4, 83.5)	64.5 (61.6, 67.3)	77.4 (76.1, 78.8)
	Female	56.9 (52.7, 61.2)	69.7 (66.3, 73.0)	70.7 (68.3, 73.1)	65.3 (63.3, 67.4)	53.9 (51.5, 56.4)	64.1 (62.8, 65.3)
Total		60.2 (57.8, 62.7)	77.0 (74.4, 79.6)	79.5 (77.6, 81.4)	72.9 (71.2, 74.5)	59.2 (57.3, 61.1)	70.9 (70.0, 71.7)

* *Keeping three valid digits*.

### Difference in consumption by region and gender grouping

As is shown in Table 4, the average intake of aquatic products and meats were 56.8 g/d and 79.0 g/d, respectively for urban residents, and 42.8 g/d and 65.8 g/d, respectively for rural residents. Urban residents consumed more aquatic products and meats than rural residents did. The difference of the consumption on crustaceans, beef, and mutton was much higher (the difference was two to five times, in same gender). And, there was only a slight difference in the consumption of fish and poultry, and almost no difference in that of pork.

**Table 4:** Daily consumption of aquatic products and meats by gender and region using 24 h recalls (g/d) (*n* = 24,106)^*^.

	**Urban**		**Rural**	
	**Male**	**Female**	**Male**	**Female**
Aquatic products
Alga	0.210 (0.140, 0.281)	0.235 (0.139, 0.330)	0.189 (0.143, 0.235)	0.203 (0.158, 0.248)
Crustaceans	16.1 (15.0, 17.2)	13.9 (12.9, 14.8)	6.23 (5.78, 6.68)	5.34 (4.89, 5.79)
Fish	43.1 (41.4, 44.8)	35.4 (34.0, 36.8)	37.5 (36.4, 38.7)	32.3 (31.2, 33.4)
Mollusks	2.95 (2.52, 3.38)	2.30 (2.00, 2.60)	2.04 (1.81, 2.26)	0.892 (0.745, 1.04)
The total 1	62.4 (60.0, 64.7)	51.8 (49.9, 53.7)	46.0 (44.5, 47.4)	39.5 (38.2, 40.9)
The total 2	56.8 (55.3, 58.3)		42.8 (41.8, 43.8)	
Meats				
Beef	10.4 (9.63, 11.2)	7.48 (6.88, 8.09)	2.83 (2.55, 3.11)	1.91 (1.69, 2.12)
Mutton	8.08 (7.22, 8.94)	5.11 (4.52, 5.69)	1.19 (0.980, 1.41)	2.03 (1.71, 2.35)
pork	52.0 (50.6, 53.5)	44.4 (42.5, 46.2)	54.1 (53.0, 55.2)	45.4 (44.5, 46.3)
poultry	17.1 (16.1, 18.2)	14.1 (13.3, 15.0)	13.7 (13.1, 14.3)	11.5 (10.9, 12.1)
The total 1	87.7 (85.3, 90.0)	71.1 (68.7, 73.4)	71.8 (70.2, 73.4)	59.7 (58.3, 61.1)
The total 2	79.0 (77.3, 80.7)		65.8 (64.8, 66.9)	

* *Keeping three valid digits*.

### Difference in consumption by SES and gender grouping

When computing SES, 7,096 subjects were excluded because of missing information, including those with no income and those who were students and attending preschools. 17,010 participants were included in the regression model, among whom 8,176 (48.1%) were male and 8,834 (51.9%) were female.

As is shown in Table 5, participants with higher SES generally consumed more meats and aquatic products than those with lower SES. However, there was no distinct difference in the consumption of alga and mollusks, with relatively low intake in general. The average intake of aquatic products for females with a low SES was lower than 40 g/d; fish consumption alone was 10 g/d less than that of people of other genders and classes. Males with high SES consumed meat more than 90 g/d, including more high-quality protein meats. Besides, women's consumption of meat lower than that of men's was mainly because of less consumption pork.

**Table 5:** Consumption of aquatic products and meats by gender and socioeconomic status (SES) grouping (*n* = 17,010)^*^.

**Gender**		**SES**					**Pairwise comparisons**
		**Low (A)**	**Median (B)**	**High (C)**	**F**	***P***	
		**Mean (95% CI)**	**Mean (95% CI)**	**Mean (95% CI)**			
Aquatic products	Male	51.6 (49.4, 53.8)	52.5 (49.8, 55.3)	63.3 (59.9, 63.7)	18.4	<0.001	C>B = A
	Female	37.3 (35.5, 39.2)	50.3 (48.2, 52.3)	57.8 (53.8, 61.8)	66.8	<0.001	C>B>A
Alga	Male	0.203 (0.133, 0.274)	0.235 (0.144, 0.327)	0.177 (0.090, 0.263)	0.391	0.675	NS^#^
	Female	0.253 (0.130, 0.375)	0.259 (0.181, 0.336)	0.232 (0.136, 0.327)	0.052	0.954	NS^#^
Crustaceans	Male	8.63 (7.83, 9.44)	10.0 (8.86, 11.2)	13.4 (11.9, 14.8)	17.7	<0.001	C>B>A
	Female	6.45 (5.61, 7.30)	9.33 (8.54, 10.1)	14.4 (12.6, 16.3)	43.6	<0.001	C>B>A
Fish	Male	40.0 (38.2, 41.8)	40.2 (38.1, 42.3)	46.9 (44.4, 49.4)	11.3	<0.001	C>B = A
	Female	28.5 (27.1, 29.9)	38.9 (37.3, 40.6)	40.3 (37.2, 43.4)	49.8	<0.001	C = B>A
Mollusks	Male	2.75 (2.37, 3.14)	2.06 (1.63, 2.48)	2.80 (2.18, 3.41)	2.98	0.051	NS^#^
	Female	2.16 (1.84, 2.48)	1.74 (1.48, 2.01)	2.84 (2.20, 3.48)	7.06	<0.001	C>B, B = A
Meats	Male	69.8 (67.5, 72.1)	78.3 (75.4, 81.3)	94.6 (90.8, 98.4)	63.8	<0.001	C>B>A
	Female	57.7 (55.7, 59.8)	63.3 (59.9, 66.7)	83.1 (79.3, 86.8)	86.2	<0.001	C>B>A
Beef	Male	2.82 (2.43, 3.21)	5.52 (4.56, 5.93)	10.0 (8.84, 11.2)	103	<0.001	C>B>A
	Female	2.47 (2.05, 2.90)	3.24 (2.81, 3.67)	9.58 (8.38, 10.8)	117	<0.001	C>B>A
Mutton	Male	2.44 (1.94, 2.94)	2.85 (2.25, 3.44)	6.38 (5.19, 7.57)	30.4	<0.001	C>B = A
	Female	1.56 (1.16, 1.97)	1.70 (1.38, 2.02)	5.56 (4.52, 6.60)	54.8	<0.001	C>B = A
Pork	Male	52.1 (50.5, 53.7)	55.9 (54.0, 57.8)	60.1 (57.7, 62.4)	16.0	<0.001	C>B>A
	Female	42.9 (41.5, 44.2)	45.2 (43.9, 46.4)	51.7 (49.3, 54.1)	23.9	<0.001	C>B>A
Poultry	Male	12.4 (11.6, 13.2)	14.3 (13.2, 15.5)	18.2 (16.6, 19.7)	24.5	<0.001	C>B>A
	Female	10.8 (10.0, 11.7)	11.7 (10.9, 12.5)	16.2 (14.7, 17.8)	23.0	<0.001	C>B = A

* *Keeping three valid digits*;

# *NS, not significant*.

## Discussion

Healthy diet patterns include nutritional food choices, appropriate consumption level and good eating style, representing the overall pattern through the long lifetime. Consuming low-quality diets tend to cause micronutrient deficiencies and contribute to a substantial rise in the incidence of diet-related obesity and non-communicable diseases. In this nationwide survey, we identified the consumption of aquatic products and meats in Chinese residents, and revealed that social, demographic and economic factors greatly affected individual dietary choices. The results indicated that the average daily consumption of all meats was 70.9 g/d across all-aged populations, which aligned with the daily recommended intake of 40–75 g/d by the DGC (21). However, the consumption of male people aged 19–60 was generally over recommended (>80 g/d). In China, the national bureau of statistics reported that the average meat intake increased from 69.9 g/d in 2000 to 102.7 g/d in 2019 for urban residents, and from 50.1g/d to 80g/d for rural residents (23). By contrast, our study reported 79.0 g/d and 65.8 g/d for urban and rural respondents, respectively. In this study, the participants included younger and elder age groups whose consumption was lower and covered different geographic regions. The larger age range of this study subjects might account for the inconsistent results.

The results showed a high frequency of consumption of red meat among males aged 19 to 44 years. Overall, these participants generally consumed red meat with more than 70g per day. Red meats like beef, pork, and lamb are rich in protein, energy, fat, and trans-fatty acid (TFA) (24). The World Cancer Research Fund advocates reducing the intake of red meat to <70 grams per day, or 500 g per week (cooked weight) (25), and avoiding processed meats such as ham, bacon, salami, hot dogs, and sausages (26). Besides, our study showed that the mean daily consumption of processed meat products was about 17.7 g/d (including barbecued meat). However, this estimate should be higher due to the presence of the recall bias. Previous research has demonstrated that an intake of 50 g/d of processed meats would increase the risk of cancer, diabetes, coronary heart disease, stroke (27–30), and even all-cause mortality (31). It is imperative to reduce the consumption of processed meat as possible. In general, the western population eats more meat than the Chinese population; A German study revealed that adult men consumed 105 grams of meat products per day and women consumed 64 grams per day in 2006 (32). In the UK, the mean daily consumption of meat products was more than 1.5 times higher than that in Germany (33). In our study, adult men consumed 80.7 grams per day, and women consumed 65.5 grams per day. The higher absolute consumption of western males in meat and meat products is comparable to that of the Western dietary pattern (34, 35). However, the difference for females is much smaller. There is a general consensus that aquatic foods are healthier than those of red meats (36–39), such as low in saturated fats and cholesterol. It was found that the mean daily consumption of aquatic products was 48.0 g, which barely meet the recommendation according to the DGC. Importantly, intake among elder people and children was insufficient (<40 g/d). The Germany food consumption survey indicated that adult men consumed 28 g/d of aquatic products and women consumed 22 g/d (32). In our study, men consumed 51.8 g/d and women consumed 44.2 g/d, which almost doubled the amount consumed by the German cohort. Considering fish is a healthier choice than the red meat, we suggest to improve more individuals to intake more fish, especially for youngster and elder. In addition, our research showed that females consumed less red meat than males did, while they ate similar quantities of white meat. Therefore, women in China appeared to make wiser food choices, and this conclusion is consistent with the German study (32).

In the study, younger and middle-aged adults taken higher level of aquatic products compared to the elderly, while children and adolescents consumed the least amount. Of these, the 31–44 age group consumed the highest amount of aquatic products. For meat consumption, younger adults and middle-aged people consumed more than 75 g/d meat per day on average and served as the main consumer group in the general population.

The results revealed that urban residents and individuals with a higher SES consumed more meat and aquatic products than did rural residents and those with a lower SES, and they mostly consumed much more beef, and mutton, and white meat such as crustaceans and poultry. In our study males with a higher SES and income generally consumed more red meat (more than 70 g/d). However, contradictory results regarding SES and dietary choices were observed in European studies people in this class, who exhibited some more comprehensive dietary preferences (40). A study from Dutch National Food Consumption Surveys suggested that dietary intake among subjects with higher SES tended to be closer to the guidelines of the Netherlands Food and Nutrition Council, and the findings were relatively stable throughout the decades assessed in the study (41). Presently, with the economic income, improved education levels and professional recognition, the pursuit of a healthy lifestyle has become more popular (42). Socio-economic and demographic factors undoubtedly contributed to dietary choices for individuals. In this study, the class, a higher SES, is related to higher red meat consumption, which seems contradictory with other studies. The reason may be the differences caused by the national dietary conditions of different countries. Unlike the western nutritional culture, the traditional Chinese diet is mainly carbohydrates, and so is the Asian diet. Since the 20^th^ century, with the popularization of the scientific diet, people have gradually realized the critical role of protein in the human body. Therefore, residents with higher SES consume more animal protein, including red and white meat. Chinese residents have gradually formed a meat consumption model dominated by pork. Of course, currently, China's economy is developing rapidly, but China is still a developing country during the period of nutrition transformation. Although residents have a deeper understanding of nutrition and have gradually realized the correlation between red meat and higher disease risk, it still takes time to change the formed eating habit, reduce red meat intake and show results. In addition, the overall food consumption pattern compensates for this seemingly contradictory result, such as personal preference, geographical availability, local ecosystems (43), price factors, etc.

This present study sheds more light on the understanding of food consumption and dietary pattern in China using a large nationwide sample size of 24,106 participants. Moreover, food consumption systems have potential effect on human health and environmental sustainability (43). Our results emphasize the need to develop policies and programs designed to monitor food consumption and assess diet nutrition among Chinese residents, preventing the incidence of various chronic diseases.

Several limitations of this study should be noticed. First, recall bias was inevitable because of the use of a retrospective dietary interview and its questionable reliability (44). Second, under-reporting, a common limitation based on history dietary assessment, could lead to measurement bias. The tendency for respondents to misreport the consumption of socially undesirable food choices has been identified in several studies in European countries (45–47). Third, the consumption of meat and aquatic products is definitely relevant to environmental factors, including organic residues and metal contaminations could not be considered due to the unavailability in the study (43). Forth, the household size was a factor influencing food intake, which was not collected in the present study. We haven't collected the details of different households but only focused on the food consumption of individuals. Moreover, the analysis by socio-economic status should be also breakdown by age to understand if a younger, adult or elder intake is more or less affected, which was not performed in this study. And the internal correlation was not analyzed as all family members were investigated the socio-economic status of the participants, which might influence the results of our study. Future studies will examine food consumption on both the individual level and household level, especially the internal correlation.

## Conclusion

Overall, the present study revealed the profile of meats and aquatic products consumption in China using a nationwide sample. Differences in consumption among different population groups (gender, age, region and SES) were reported. Understanding the trends and determinants of dietary intake could help build and maintain healthy diet patterns in China. Further researches based on dynamic dietary profiles should be conducted to give top priority to suitable food choice and healthy diet patterns for the general population.

## Data availability statement

The original contributions presented in the study are included in the article/supplementary materials, further inquiries can be directed to the corresponding author/s.

## Ethics statement

The studies involving human participants were reviewed and approved by Ethics Committee of the China Center for Disease Control and Prevention. Written informed consent to participate in this study was provided by the participants' legal guardian/next of kin.

## Author contributions

AL and LJ designed the study. QW drafted the protocol, analyzed data, and wrote the manuscript. SL, HW, and CS participated in the data collection and analyzed and discussed the results. All authors have reviewed final manuscript and approved it for publication.

## Conflict of interest

The authors declare that the research was conducted in the absence of any commercial or financial relationships that could be construed as a potential conflict of interest. The reviewer ZB declared a shared affiliation, with no collaboration, with the authors HW and CS to the handling editor at the time of the review.

## Publisher's note

All claims expressed in this article are solely those of the authors and do not necessarily represent those of their affiliated organizations, or those of the publisher, the editors and the reviewers. Any product that may be evaluated in this article, or claim that may be made by its manufacturer, is not guaranteed or endorsed by the publisher.

## Abbreviations

DGC: Dietary Guidelines for Chinese Residents
NBS: China's national bureau of statistics
SES: socioeconomic status
CFCS: Chinese Food Consumption Survey
NFSA: China National Center for Food Safety Risk Assessment
CDC: Chinese Center for Disease Control and Prevention
PPS: population-proportional sampling
FFQ: food frequency questionnaire
SEI: social class-index
CI: confidence interval
ANOVA: one-way classification analysis of variance
TFA: trans-fatty acid.

## References

[1] Archundia Herrera MC Subhan FB Chan CB. Dietary patterns and cardiovascular disease risk in people with type 2 diabetes. Curr Obes Rep. (2017) 6:405–13. 10.1007/s13679-017-0284-5 29063379

[2] Grosso G Bella F Godos J Sciacca S Del Rio D Ray S. Possible role of diet in cancer: systematic review and multiple meta-analyses of dietary patterns, lifestyle factors, and cancer risk. Nutr Rev. (2017) 75:405–19. 10.1093/nutrit/nux012 28969358

[3] Jannasch F Kroger J Schulze MB. Dietary patterns and type 2 diabetes: a systematic literature review and meta-analysis of prospective studies. J Nutr. (2017) 147:1174–82. 10.3945/jn.116.242552 28424256

[4] Smethers AD Rolls BJ. Dietary management of obesity: cornerstones of healthy eating patterns. Med Clin North Am. (2018) 102:107–24. 10.1016/j.mcna.2017.08.009 29156179 PMC5726407

[5] Mohan V Unnikrishnan R Shobana S Malavika M Anjana RM. Sudha V. Are excess carbohydrates the main link to diabetes & its complications in Asians? Indian J Med Res. (2018) 148:531–8. 10.4103/ijmr.IJMR_1698_18 30666980 PMC6366262

[6] Fayet-Moore F Baghurst K Meyer BJ. Four models including fish, seafood, red meat and enriched foods to achieve Australian dietary recommendations for n-3 LCPUFA for all life-stages. Nutrients. (2015) 7:8602–14. 10.3390/nu7105413 26492269 PMC4632433

[7] Sans P Combris P. World meat consumption patterns: an overview of the last fifty years (1961–2011). Meat Sci. (2015) 109:106–11. 10.1016/j.meatsci.2015.05.012 26117396

[8] Wagner MG Rhee Y Honrath K Blodgett Salafia EH Terbizan D. Nutrition education effective in increasing fruit and vegetable consumption among overweight and obese adults. Appetite. (2016) 100:94–101. 10.1016/j.appet.2016.02.002 26850310

[9] Ruxton C. The role of red meat in a balanced diet. Nurs Stand. (2011) 26:41–8. 10.7748/ns2011.10.26.7.41.c8759 23256256

[10] Qing GZ Lu Y Yi T Zhang KQ Tang ZH. The relationship of frequency of meat consumption and osteoporosis in Chinese postmenopausal women. Int J Clin Exp Med. (2015) 8:21130–7. 26885045 PMC4723890

[11] Schwingshackl L Schwedhelm C Hoffmann G Knuppel S Iqbal K Andriolo V. Food groups and risk of hypertension: a systematic review and dose-response meta-analysis of prospective studies. Adv Nutr. (2017) 8:793–803. 10.3945/an.117.017178 29141965 PMC5683007

[12] Ghanbari B Khaleghparast S Ghadrdoost B Bakhshandeh H. Nutritional status and coronary artery disease: a cross sectional study. Iran Red Crescent Med J. (2014) 16:e13841. 10.5812/ircmj.13841 24829768 PMC4005430

[13] Pan A Sun Q Bernstein AM Schulze MB Manson JE Stampfer MJ. Red meat consumption and mortality: results from 2 prospective cohort studies. Arch Intern Med. (2012) 172:555–63. 10.1001/archinternmed.2011.2287 22412075 PMC3712342

[14] Abid Z Cross AJ Sinha R. Meat, dairy, and cancer. Am J Clin Nutr. (2014) 100:386S−93. 10.3945/ajcn.113.071597 24847855 PMC4144110

[15] Kim K Hyeon J Lee SA Kwon SO Lee H Keum N. Role of total, red, processed, and white meat consumption in stroke incidence and mortality: a systematic review and meta-analysis of prospective cohort studies. J Am Heart Assoc. (2017) 6:e005983. 10.1161/JAHA.117.005983 28855166 PMC5634267

[16] Cantoral A Batis C Basu N. National estimation of seafood consumption in Mexico: implications for exposure to methylmercury and polyunsaturated fatty acids. Chemosphere. (2017) 174:289–96. 10.1016/j.chemosphere.2017.01.109 28183054

[17] Brownie S Muggleston H Oliver C. The 2013 Australian dietary guidelines and recommendations for older Australians. Aust Fam Physician. (2015) 44:311–5. 26042404

[18] Lee JH Duster M Roberts T Devinsky O. United States dietary trends since 1800: lack of association between saturated fatty acid consumption and non-communicable diseases. Front Nutr. (2022) 8:748847. 10.3389/fnut.2021.748847 35118102 PMC8805510

[19] Walrabenstein W de Jonge CS Kretova AM. Commentary: United States dietary trends since 1800: lack of association between saturated fatty acid consumption and non-communicable diseases. Front Nutr. (2022) 9:891792. 10.3389/fnut.2022.891792 35571907 PMC9096699

[20] DeSalvo KB Olson R Casavale KO. Dietary guidelines for Americans. JAMA. (2016) 315:457–8. 10.1001/jama.2015.18396 26746707

[21] Wang SS Lay S Yu HN Shen SR. Dietary guidelines for Chinese residents (2016): comments and comparisons. J Zhejiang Univ Sci B. (2016) 17:649–56. 10.1631/jzus.B1600341 27604857 PMC5018612

[22] Ren CR. Measurement methodology on social economic status index of students. J Educ Stud. (2010) 6:77–82. 10.14082/j.cnki.1673-1298.2010.05.010

[23] Cui C Wang Ml. China's meat consumption development and its prospect. Agric Outlook. (2016) 12:74–80.

[24] Bendsen NT Christensen R Bartels EM Astrup A. Consumption of industrial and ruminant trans fatty acids and risk of coronary heart disease: a systematic review and meta-analysis of cohort studies. Eur J Clin Nutr. (2011) 65:773–83. 10.1038/ejcn.2011.34 21427742

[25] Clonan A Wilson P Swift JA Leibovici DG Holdsworth M. Red and processed meat consumption and purchasing behaviours and attitudes: impacts for human health, animal welfare and environmental sustainability. Public Health Nutr. (2015) 18:2446–56. 10.1017/S1368980015000567 25766000 PMC10271490

[26] Larsson SC Orsini N. Red meat and processed meat consumption and all-cause mortality: a meta-analysis. Am J Epidemiol. (2014) 179:282–9. 10.1093/aje/kwt261 24148709

[27] Guo J Wei W Zhan L. Red and processed meat intake and risk of breast cancer: a meta-analysis of prospective studies. Breast Cancer Res Treat. (2015) 151:191–8. 10.1007/s10549-015-3380-9 25893586

[28] Micha R Michas G Mozaffarian D. Unprocessed red and processed meats and risk of coronary artery disease and type 2 diabetes–an updated review of the evidence. Curr Atheroscler Rep. (2012) 14:515–24. 10.1007/s11883-012-0282-8 23001745 PMC3483430

[29] Kaluza J Wolk A Larsson SC. Red meat consumption and risk of stroke: a meta-analysis of prospective studies. Stroke. (2012) 43:2556–60. 10.1161/STROKEAHA.112.663286 22851546

[30] Chan DS Lau R Aune D Vieira R Greenwood DC Kampman E. Red and processed meat and colorectal cancer incidence: meta-analysis of prospective studies. PLoS ONE. (2011) 6:e20456. 10.1371/journal.pone.0020456 21674008 PMC3108955

[31] Abete I Romaguera D Vieira AR Lopez de. Munain A, Norat T. Association between total, processed, red and white meat consumption and all-cause, CVD and IHD mortality: a meta-analysis of cohort studies. Br J Nutr. (2014) 112:762–75. 10.1017/S000711451400124X 24932617

[32] Heuer T Krems C Moon K Brombach C Hoffmann I. Food consumption of adults in Germany: results of the German national nutrition survey ii based on diet history interviews. Br J Nutr. (2015) 113:1603–14. 10.1017/S0007114515000744 25866161 PMC4462161

[33] Whitton C Nicholson SK Roberts C Prynne CJ Pot GK. Olson A. National Diet and Nutrition Survey: UK food consumption and nutrient intakes from the first year of the rolling programme and comparisons with previous surveys. Br J Nutr. (2011) 106:1899–914. 10.1017/S0007114511002340 21736781 PMC3328127

[34] Naja F Hwalla N Itani L Karam S Sibai AM Nasreddine L. Western dietary pattern is associated with overweight and obesity in a national sample of Lebanese adolescents (13–19 years): a cross-sectional study. Br J Nutr. (2015) 114:1909–19. 10.1017/S0007114515003657 26431469 PMC4635384

[35] Hariharan D Vellanki K Kramer H. The western diet and chronic kidney disease. Curr Hypertens Rep. (2015) 17:16. 10.1007/s11906-014-0529-6 25754321

[36] Schmidt JA Crowe FL Appleby PN Key TJ Travis RC. Serum uric acid concentrations in meat eaters, fish eaters, vegetarians and vegans: a cross-sectional analysis in the EPIC-Oxford cohort. PLoS ONE. (2013) 8:e56339. 10.1371/journal.pone.0056339 23418557 PMC3572016

[37] Joosen AM Lecommandeur E Kuhnle GG Aspinall SM Kap L Rodwell SA. Effect of dietary meat and fish on endogenous nitrosation, inflammation, and genotoxicity of faecal water. Mutagenesis. (2010) 25:243–7. 10.1093/mutage/gep070 20106932

[38] Rohrmann S Linseisen J Nothlings U Overvad K Egeberg R Tjonneland A. Meat and fish consumption and risk of pancreatic cancer: results from the European prospective investigation into cancer and nutrition. Int J Cancer. (2013) 132:617–24. 10.1002/ijc.27637 22610753

[39] Okubo H Sasaki S Murakami K Takahashi Y. Freshmen in dietetic courses study IIg. The ratio of fish to meat in the diet is positively associated with favorable intake of food groups and nutrients among young Japanese women. Nutr Res. (2011) 31:169–77. 10.1016/j.nutres.2011.02.005 21481710

[40] Clonan A Roberts KE Holdsworth M. Socioeconomic and demographic drivers of red and processed meat consumption: implications for health and environmental sustainability. Proc Nutr Soc. (2016) 75:367–73. 10.1017/S0029665116000100 27021468 PMC4974628

[41] Hulshof KF Brussaard JH Kruizinga AG Telman J Lowik MR. Socio-economic status, dietary intake and 10 y trends: the Dutch national food consumption survey. Eur J Clin Nutr. (2003) 57:128–37. 10.1038/sj.ejcn.1601503 12548307

[42] Humphries DL Behrman JR Crookston BT Dearden KA Schott W Penny ME. Households across all income quintiles, especially the poorest, increased animal source food expenditures substantially during recent Peruvian economic growth. PLoS ONE. (2014) 9:e110961. 10.1371/journal.pone.0110961 25372596 PMC4220962

[43] Willett W Rockström J Loken B. Food in the anthropocene: the EAT-Lancet commission on healthy diets from sustainable food systems. Lancet. (2019) 393:447–92. 10.1016/S0140-6736(18)31788-4 30660336

[44] St George SM Van Horn ML Lawman HG Wilson DK. Reliability of 24-H dietary recalls as a measure of diet in African-American youth. J Acad Nutr Diet. (2016) 116:1551–9. 10.1016/j.jand.2016.05.011 27394936 PMC5039054

[45] Dubuisson C Lioret S Touvier M Dufour A Calamassi-Tran G Volatier JL. Trends in food and nutritional intakes of French adults from 1999 to 2007: results from the INCA surveys. Br J Nutr. (2010) 103:1035–48. 10.1017/S0007114509992625 20028601

[46] Vandevijvere S De Vriese S Huybrechts I Moreau M Temme E De Henauw S. The gap between food-based dietary guidelines and usual food consumption in Belgium, 2004. Public Health Nutr. (2009) 12:423–31. 10.1017/S1368980008002164 18426635

[47] Leclercq C Arcella D Piccinelli R Sette S Le Donne C Turrini A. The Italian National Food Consumption Survey INRAN-SCAI 2005-06: main results in terms of food consumption. Public Health Nutr. (2009) 12:2504–32. 10.1017/S1368980009005035 19278564

